# Health-related quality of life and scar satisfaction in a cohort of children operated on for sacrococcygeal teratoma

**DOI:** 10.1186/s12955-020-01350-y

**Published:** 2020-04-17

**Authors:** Mette Hambraeus, Lars Hagander, Einar Arnbjörnsson, Anna Börjesson, Pernilla Stenström

**Affiliations:** 1 Department of Pediatric surgery, Skane University Hospital Lund, Lasarettgatan 48, 222 41 Lund, Sweden; 2 Skane University Hospital, Department of Clinical Sciences Lund, Pediatric Surgery, Lund University, Lund, Sweden

**Keywords:** Sacrococcygeal teratoma, Health-related quality of life, Scar, Children

## Abstract

**Aim:**

The aims of this study were to evaluate health-related quality of life (HRQoL) in children with sacrococcygeal teratoma and to explore the effect of the scar on physical, emotional and behavioral aspects.

**Methods:**

A cohort of children operated on for sacrococcygeal teratoma between 2000 and 2013 at Lund University Hospital, Sweden, and their parents were interviewed. HRQoL was evaluated with PedsQL, and scar satisfaction was estimated through Patient Observer Scar Assessment Score (POSA).

**Results:**

All eligible children (*n* = 17) were included (100% response rate). Median age was 7.3 years (range 3.5–16.0). Mean total PedsQL score was 92.3 (range 72.0 to 99.0). Patients with comorbidity scored lower (87.5) than those without (95.0) (*p* < 0.05). Pain during sitting down was reported by two (20%) patients, and itching was reported by another two patients (20%) aged > 8 years. No children reported that they avoided situations due to the scar, and most (80% of children and 90% of parents) reported absent or only mild negative emotions when considering the scar.

**Conclusion:**

Children with sacrococcygeal teratoma had a good overall HRQoL, but comorbidity reduced the outcome. A few children reported scar-related impact on physical, behavioral and emotional aspects.

## Introduction

Sacrococcygeal teratomas are rare congenital tumors with a birth prevalence of 1:10,000–1:40,000. The tumors are often diagnosed prenatally and may grow to enormous proportions with a risk of developing fetal hydrops and intrauterine demise [1–5]. Prenatal compression of surrounding pelvic structures and surgical trauma contribute to the well-described complication profile including urinary tract and bowel dysfunction [2, 6, 7]. In addition to functional considerations, children with sacrococcygeal teratomas often have a distinct scar and irregular buttock contour caused by the tumor’s fragmentation of the gluteal musculature.

Health-related quality of life (HRQoL) is defined as a person’s individual perception of a condition’s impact on physical, mental and social aspects of life [8]. HRQoL has received increasing attention within pediatric specialties in recent decades, and it is a common concern to parents who are expecting a child with sacrococcygeal teratoma. Although transient problems with micturition and defecation may occur in otherwise healthy children, complications following sacrococcygeal teratoma often constitute a chronic condition with a potential to reduce HRQoL. Previous studies have found reduced HRQoL in children with urinary tract and bowel problems [9–11], which may imply that children born with sacrococcygeal teratoma are subjected to similar risks. A Dutch study evaluating adults born with sacrococcygeal teratoma showed no significant reduction in HRQoL [12], but no study has hitherto evaluated the impact on HRQoL during the course of childhood. Increased knowledge concerning aspects influencing HRQoL in patients born with sacrococcygeal teratoma would contribute to an evidence-based risk stratification of patients in early childhood, and this could potentially direct medical and psychological interventions towards improved long-term outcome. Therefore, this study aimed to evaluate HRQoL in children with sacrococcygeal teratoma, as well as to explore the effect of the postoperative scar on physical, emotional and behavioral aspects.

## Methods

### Data collection

We conducted a cross-sectional study of all surviving children operated on for sacrococcygeal teratoma during 2000–2013 at our tertiary center of pediatric surgery in Lund, Sweden. The interviews were carried out through 2016–2017 and the children were invited to an appointment at the children’s hospital to convey this study. The patients and at least one parent were invited to the Children’s Hospital to complete a questionnaire-based interview concerning HRQoL and scar satisfaction. A researcher not directly involved in the care of the patients conducted the interviews through asking the questions according to the questionnaires while clarifying uncertainties.

The cohort has previously been evaluated for prenatal growth pattern [4], and urinary tract/bowel symptoms at long-term follow up [6, 13]. The results of the latter study were based upon data collected during the same interview setting and were integrated in the present study through comparing HRQoL in the children with and without reduced urinary/bowel function and comorbidites. No further socio-economic factors were evaluated.

### Instruments

HRQoL was evaluated through the PedsQL 4.0 generic core scale, a validated questionnaire addressing children aged 2–18 years [14]. The questionnaire contains 23 items covering physical, emotional, social and school functioning. A psychosocial health summary score is based on an average of emotional, social and school functioning. The patients were asked to recall the past month and answer from 0 (never a problem) to 4 (almost always a problem). These scores were transformed into a 5-point Likert scale from 0 = 100%, 1 = 75%, 2 = 50%, 3 = 25%, 4 = 0%, where 100% corresponds to the highest possible HRQoL-score. Different versions of the questionnaire were based upon developmentally appropriate age groups, i.e. 2–4, 5–7, 8–12, 13–18 years. Through the help of the interviewer, children aged 5 years and above answered the questions themselves, while a parent proxy questionnaire was used for children aged 2–4 years.

The parents’ subjective opinion concerning scar appearance and symptoms was evaluated through the patient module of The Patient Observer Scar Assessment Score (POSAS) [15]. The POSAS was introduced in 2004 and aims to measure the quality of scar tissue through a patient and observer module. The questionnaire has previously been used as a proxy model to describe scars in children [16, 17]. No observer evaluation of the physical appearance could be performed due to lack of ethical approval, and consequently only the patient module was used. The patient module of the POSAS evaluates experienced pain, pruritus, color, stiffness, thickness and regularity compared to normal skin. Each parameter is scored from 1 (as normal skin) to 10 (very unlike normal skin), yielding a total score ranging from 6 to 60, with 6 being the most advantageous outcome.

Children aged 8 years and above (*n* = 10) and available parents of all included children (*n* = 32) were asked independently to complete questions about behavioral and emotional aspects concerning the scar. The parents answered the questionnaires independently, while the interviewer helped the child when necessary. When only one parent could attend the visit at the children’s hospital, a questionnaire filled in by the second parent (if applicable) was posted to the hospital after the interview.

### Statistical analysis

PedsQL-scores in subgroups were described by median [range], and Mann-Whitney U-test was used to compare PedsQL-scores. Spearman correlation was used to analyze the relationship between PedsQL and POSA scores. Results were analyzed using IBM SPSS Statistics, version 24.0, Armonk, NY; IBM Corp.

## Results

All eligible children (*n* = 17) were included in the study, yielding a response rate of 100%. At the time of the questionnaire the children had a median age of 7.3 years (range 3.5–16.0), and the girl:boy ratio was 3.25:1. The functional results and tumor characteristics have been reported previously [6]: A total of 29% had neurogenic lesions of the bladder, 24% required medical treatment of constipation and 29% had problems with soiling. Two patients reported fecal incontinence – one girl reported occasional problems, and one girl was treated successfully with antegrade enemas through an appendicostomy. Four children had comorbidities: Beckwith-Wiedeman syndrome, familial hypercalciuria, attention deficit hyperactivity disorder (ADHD) and general anxiety. The remaining 76% of the cohort had no other diagnoses.

### Health-related quality of life

The PedsQL 4.0 core instrument was completed for five children aged 2–4 years (by proxy), five children aged 5–7 years, five children aged 8–12 years and two children aged 13–18 years. HRQoL results are displayed in Table 1. The median total PedsQL score was 94 ranging from 72 to 99. The median psychosocial health summary score was 92, with high scores within all subcategories consisting of physical, social and school functioning. The proxy scores of young children aged 2–4 years did not differ significantly from those of older children completing self-report questionnaires.

**Table 1:** Health related quality of life scores (PedsQL) in patients with sacrococcygeal teratoma

	Median	Range
Total score	94	72–99
Physical score	97	78–100
Psychosocial health	92	79–98
Emotional Functioning	90	45–100
Social Functioning	100	70–100
School Functioning	95	60–100

Five children were diagnosed with neurogenic bladder/bowel, of whom three children used self-catheterization to empty the bladder. These children did not report significantly altered HRQoL compared to children with normal urinary tract/bowel function (Table 2).

**Table 2:** Health related quality of life scores (PedsQL) in sacrococcygeal teratoma patients with neurogenic bladder and/or neurogenic bowel function compared to patients with normal bladder and bowel function

	Neurogenic bladder/bowel function (***N*** = 5)		Normal bladder/bowel function (***N*** = 12)		
	Median	Range	Median	Range	*P*
Total score	93.0	72–97	95.0	83–99	*0.19*^*a*^
Physical score	94.0	78–100	97.0	91–100	*0.83*^*a*^
Psychosocial health	94.0	68–97	92.0	78–98	*0.56*^*a*^
Emotional functioning	90.0	75–100	90.0	45–100	*0.75*^*a*^
Social functioning	100.0	70–100	100.0	90–100	*0.71*^*a*^
School functioning	90.0	60–95	100.0	75–100	*0.07*^*a*^

^a^ Mann-Whitney U-test

A comparison of children with and without comorbidities is presented in Table 3. The four children with comorbidities had significantly lower PedsQL overall score, physical score and school functioning score. The two children with ADHD and general anxiety disorder had lowest HRQoL, with total scores of 72.0 and 83.0 respectively.

**Table 3:** Health related quality of life scores (PedsQL) in sacrococcygeal teratoma patients with comorbidities^a^ compared to patients without comorbidities

	Comorbidities (***N*** = 4)		No comorbidities (***N*** = 13)		
	Median	Range	Median	Range	*P*
Total score	87.5	72–94	95.0	86–99	*< 0.05*^*b*^
Physical score	87.5	78–94	97.0	91–100	*< 0.01*^*b*^
Psychosocial health	86.0	68–96	92.0	79–98	*0.35*^*b*^
Emotional functioning	82.5	65–100	90.0	45–100	*0.70*^*b*^
Social functioning	95.0	70–100	100.0	90–100	*0.20*^*b*^
School functioning	81.5	60–92	95.0	75–100	*< 0.05*^*b*^

^a^ Beckwith-Wiedeman, familial hypercalciuria, ADHD, general anxiety disorder

^b^ Mann-Whitney U-test

### Implications of the scar

**Fig. 1:**
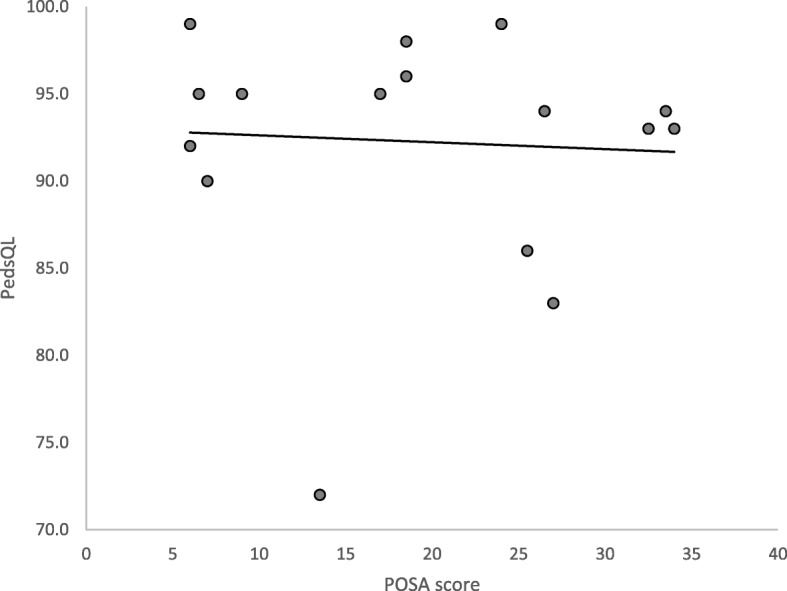
Correlation between the children’s PedsQL-score (0–100) and parents’ average POSA score (6–60, with high score indicating poor outcome). *N* = 17. Spearman correlation: *p* = 0.37

Questionnaires regarding the postoperative scar were answered by patients aged 8 years and above (*n* = 10) and all available parents of the total cohort (*n* = 32). Two patients had scar revision performed – one at age 10 due to cosmetic considerations, the other at 1 year of age due to asymmetry and pain while sitting. There was no significant difference in how mothers and fathers experienced their child’s scar. Mothers reported a median POSA score of 13.5 (6–36), and fathers 18.5 (6–47) (*p* = 0.93). Figure 1 displays a plot of the correlation between PedsQL score and parents’ POSA score. Spearman rank order test showed no significant correlation between the parameters (*p* = 0.37).

**Fig. 2:**
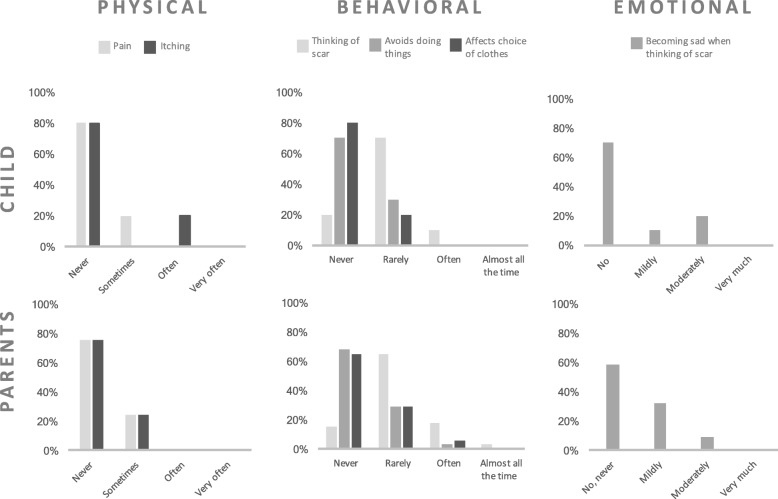
Reports of physical, behavioral and emotional aspects of the scar. Comparison of children with sacrococcygeal teratoma (*n* = 10) and their parents (*n* = 32)

A distribution of children’s and parents’ answers regarding physical symptoms, behavioral and emotional aspects is presented in Fig. 2. Physical symptoms related to the scar at the time of follow-up were uncommon in the cohort. Occasional pain during long periods of sitting down was reported by two (20%) patients, and itching was a problem for two (20%) other patients. The majority of children and parents thought of the scar only rarely or not at all. Frequent thoughts concerning the scar were reported by one child and by 18% of the parents. One mother described having negative thoughts of the scar several times every day, while one father described feelings of gratitude when seeing or thinking of the scar, since this reminded him of how lucky the family had been.

Both child and proxy reports showed that 70% of the children never and 30% rarely avoided situations due to the scar. No children experienced consistently evading behavior, neither self or proxy-reported. Two children avoided wearing certain types of tight clothes due to the scar, but only on rare occasions. The vast majority of children and parents reported absent or mild negative emotions when thinking of the scar, while 20% of the children and 10% of parents were moderately sad when considering the scar. Many children attributed the low level of distress to the discrete location of the scar, being concealed in most situations.

## Discussion

This study showed that children born with sacrococcygeal teratoma had a very good overall HRQoL. Physical symptoms and behavioral/emotional consequences were uncommon in the cohort, and we found no correlation between parents’ assessment of the scar and the HRQoL of the child.

The results showing a good overall HRQoL in this patient group correlate well with the study by Shalaby et al. presenting low levels of appearance-related distress in adults with sacrococcygeal teratoma [18], and a study by Kremer et al. reporting normal HRQoL among adults operated on for sacrococcygeal teratoma during childhood [12]. The HRQoL of healthy Swedish children has not been analyzed using the PedsQL 4.0 generic core scale. The original validation study of the PedsQL questionnaire analyzed healthy American children with a mean age of 9.3 years, and found a mean total PedsQL score of 83.0, a physical score of 84.4 and a psychosocial health score of 82.4 [14]. These PedsQL-scores seem to be inferior to those of the children with sacrococcygeal teratoma included in this study (Table 1), although no statistical comparison can be made due to the small number of children analyzed in the present study.

Children with urinary tract/bowel dysfunction had HRQoL scores equivalent to those of sacrococcygeal teratoma patients without functional problems. This is somewhat surprising considering several authors reporting lowered self-esteem among children with urinary incontinence [10] and constipation [11]. Bongers et al. found a high impact on emotional functioning in children with constipation and fecal incontinence, while social function was less affected [9]. The two children with fecal incontinence in our study were only 4 and 5 years old at the time of the study; according to a study by Grano et al. increasing impact on HRQoL may occur with age [19].

Children with comorbidities scored significantly lower on physical and school functioning, especially two children with ADHD and general anxiety disorder. This indicates that HRQoL among children with sacrococcygeal teratoma is more contingent upon comorbidities than the teratoma per se. Several previous studies have reported reduced HRQoL in children with ADHD [20], but to the authors’ best knowledge, no studies have described increased prevalence of ADHD among children operated on for sacrococcygeal teratoma. However, a possible causative link between the diagnoses could be the increased risk of very premature birth associated with large sacrococcygeal teratoma [21, 22], which has been correlated to higher levels of ADHD and autism spectrum diagnoses [23].

In general, generic HRQoL instruments have a lower sensitivity to detect reduced health-related quality of life within a specific diagnosis, and especially in congenital diseases, when compared to disease-specific instruments [8]. No disease-specific HRQoL instrument exists for sacrococcygeal teratoma, and the generic nature of the questionnaire may partly explain the good self-reported HRQoL compared with healthy children. Generic psychosocial scores approaching normal values have been found by some authors in other chronic childhood illnesses, i.e. cystic fibrosis [24] and diabetes [25]. Coping strategies in children with chronic diagnoses are well studied [26], and may explain differences in HRQoL regardless of comparable symptoms.

### Physical, emotional and behavioral aspects of the scar

The parents’ overall assessment of the scar was located in the lower third of the POSA scale, expressing a good overall satisfaction with the cosmetic outcome. The vast majority of children and parents thought very little of the scar, and comments during the interviews indicated that children and parents were grateful for the discrete location of the scar. This information is important to convey in the prenatal and early postoperative phase, when parents often worry about the cosmetic appearance of the scar, and what this may entail for the future of the child.

A Dutch long-term follow-up study by Derikx et al. [2] showed a high frequency of dissatisfaction with the scar following sacrococcygeal teratoma resection, with 40.3% reporting a cosmetically unacceptable result. Dissatisfaction was found to be correlated to large tumor size and early diagnosis of the teratoma. The included patients were somewhat older than our cohort, up to 22 years of age, and it is possible that dissatisfaction increases in adolescence and early adulthood. In addition, cultural differences may partly explain the heterogeneity in outcome concerning appearance-related satisfaction and HRQoL.

## Limitations

Due to the rarity of the disorder the number of included patients in the cohort is small, which limits conclusions being drawn from the study. We used a generic HRQoL instrument, which enables comparison with healthy children and other diagnostic categories. However, generic instruments reduce sensitivity, and construction of a validated questionnaire for sacrococcygeal teratoma, including bladder, bowel and scar aspects, could possibly further elucidate problems concerning HRQoL. Future studies should evaluate whether children with comorbidities, especially neuropsychiatric disorders, have a reduced capability to cope with functional complications following sacrococcygeal teratoma. These children could then be identified early in order to increase self-efficacy, reduce stress and endorse adaptive, problem-solving coping strategies. Further, our study group is young and problems concerning both cosmetics and reduced HRQoL due to micturition/bowel issues may emerge during the course of adolescence and early adulthood. In addition, the time prolapsed since surgery varies in the study group which could possibly impact the outcome. Longitudinal studies following the children until the third decade of life could potentially help shed light on the dynamics of HRQoL in this patient group.

## Conclusion

In conclusion, according to generic quantitative measurements, children with sacrococcygeal teratoma had a good overall HRQoL during childhood. Children with a comorbidity had reduced outcome regarding HRQoL. Few children had physical symptoms concerning their scar, and scar-related impact on behavior or emotions was generally very low. The present findings are useful in the prenatal and postoperative setting, when the child’s future and general HRQoL is of paramount concern to the parents.

## Data Availability

The datasets used and/or analyzed during the current study are available from the corresponding author on reasonable request.

## Abbreviations

HRQoL: Health-related quality of life
POSAS: Patient observer scar assessment score
ADHD: Attention deficit hyperactivity disorder
